# Guidance for the utility of linear models in meta-analysis of genetic association studies of binary phenotypes

**DOI:** 10.1038/ejhg.2016.150

**Published:** 2016-11-16

**Authors:** James P Cook, Anubha Mahajan, Andrew P Morris

**Affiliations:** 1 Department of Biostatistics, University of Liverpool, Liverpool, UK; 2 Wellcome Trust Centre for Human Genetics, University of Oxford, Oxford, UK

## Abstract

Linear mixed models are increasingly used for the analysis of genome-wide association studies (GWAS) of binary phenotypes because they can efficiently and robustly account for population stratification and relatedness through inclusion of random effects for a genetic relationship matrix. However, the utility of linear (mixed) models in the context of meta-analysis of GWAS of binary phenotypes has not been previously explored. In this investigation, we present simulations to compare the performance of linear and logistic regression models under alternative weighting schemes in a fixed-effects meta-analysis framework, considering designs that incorporate variable case–control imbalance, confounding factors and population stratification. Our results demonstrate that linear models can be used for meta-analysis of GWAS of binary phenotypes, without loss of power, even in the presence of extreme case–control imbalance, provided that one of the following schemes is used: (i) effective sample size weighting of *Z*-scores or (ii) inverse-variance weighting of allelic effect sizes after conversion onto the log-odds scale. Our conclusions thus provide essential recommendations for the development of robust protocols for meta-analysis of binary phenotypes with linear models.

## Introduction

Linear mixed models (LMMs) have received increasing prominence in the analysis of genome-wide association studies (GWAS) of complex human traits because they account for genetic structure, across participants, which arises from population stratification, cryptic relatedness or close familial relationships.^1, 2, 3, 4, 5, 6, 7^ In this framework, structure is modelled by means of a genetic relationship matrix (GRM), constructed from genome-wide SNP genotype data across study participants (or from known familial relationships). A random-effects model is then used to evaluate the evidence of association for an SNP by accounting for the contribution of the GRM to the overall variance of the trait. This flexible modelling framework can incorporate fixed effects to account for covariates, and can be used to estimate components of heritability that are explained by (subsets of) genotyped SNPs.^8, 9^

Linear models assume that the outcome of interest is a quantitative trait with a Gaussian distribution. However, it has become increasingly common to use LMM approaches in population- and family-based GWAS of binary phenotypes because of their flexibility in accounting for structure, and their computational tractability in comparison with logistic mixed models. Linear models have the disadvantage that allelic effect estimates cannot be interpreted, directly, in terms of the odds ratio (OR), although approximations on the log-odds scale can be obtained.^10^ Recent studies have also demonstrated that LMMs have less power than traditional logistic regression modelling techniques in GWAS of case–control phenotypes unless ascertainment is adequately accounted for.^11, 12^

While the properties of linear (mixed) models in the analysis of GWAS of binary phenotypes *at the cohort level* have been explored previously,^10^ their utility in the context of *meta-analysis* has not been investigated. In this study, therefore, we present simulations to compare the type I error rates and power of generalised linear (mixed) models under alternative weighting schemes in a fixed-effects meta-analysis framework. We consider a range of study designs that incorporate variable case–control imbalance across GWAS to reflect the increasing use of large-scale, population-based biobanks, and investigate the impact of confounders and population stratification on the properties of the analytical strategies. We conclude by making recommendations for the development of robust protocols for meta-analysis of GWAS of binary phenotypes with linear (mixed) models, which will be highly relevant in the era of large-scale consortium efforts to unravel the genetic basis of complex human diseases.

## Materials and methods

Consider a GWAS of *n* participants, with binary phenotypes, genome-wide genotypes and additional covariates denoted by **y**, **G** and **x**, respectively. We denote the phenotype of the *i*th participant by *y*_*i*_∈{0, 1}, and their genotype at the *j*th SNP by *G*_*ij*_∈[0, 2], coded under a dosage model in the number of minor alleles. In a generalised linear mixed modelling framework,


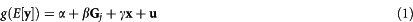


where *g*(.) is the link function, *β* is the allelic effect of the *j*th SNP on the phenotype and *γ* is a vector of covariate regression parameters. In this expression, **u** is a vector of random effects, defined by **u**~MVN(0, *λ***K**), for the variance component *λ* and GRM **K**, derived from genome-wide SNP data (or known familial relationships) to account for population structure. A likelihood ratio test with one degree of freedom is then formed by comparing the maximised log likelihood of the unconstrained model (1) with that obtained under the null hypothesis of no association, *β*=0. Note that model (1) reduces to a generalised linear model (no random effects) for *λ*=0, which is appropriate in the absence of structure because of population stratification and/or familial relationships.

Under a logistic regression model, for the logit link function, the maximum-likelihood estimate of the allelic effect, 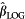, can be interpreted directly as the log-OR of the *j*th SNP. However, under a linear regression model, for the identity link function, the maximum-likelihood estimate of the allelic effect, 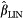, is measured on the wrong scale. Nevertheless, we can obtain an approximation of the allelic log-OR and corresponding variance from the linear model,^10^ given by


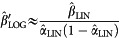


and


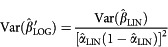


where 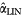 is the maximum-likelihood estimate of the intercept. In practice, 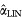 is usually obtained from the null model for which *β*_LIN_=0, because the effect of any SNP on the phenotype is expected to be small. Here, we estimate 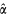 by the proportion of participants that are cases, for which the correction factor 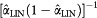 is minimised when the number of cases and controls in the study is equal (ie, no imbalance). This transformation of parameter estimates from the linear regression model has been demonstrated to provide an accurate approximation of the allelic log-OR provided that genetic effects are small, the case–control ratio is well balanced and the SNP is common.^10^

### Fixed-effects meta-analysis

Consider *N* GWAS, for which we have tested for association of the phenotype with the *j*th SNP under a generalised linear model (1). We denote the *effective* sample size of the *k*th GWAS by *n*_*k*_, given by


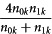


where *n*_0*k*_ and *n*_1*k*_ denote the number of controls and cases, respectively. In the *k*th GWAS, we also denote the *P*-value obtained from the regression model by *p*_*k*_, and the estimated allelic effect from the regression model by 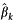.

Under an effective sample size weighting scheme, we obtain a combined *Z*-score for association of the *j*th SNP across GWAS by


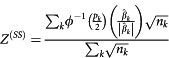


where *φ*^−1^ is the inverse normal distribution function. Alternatively, under an inverse-variance weighting scheme, we obtain an estimate of the allelic effect of the *j*th SNP on the phenotype, and the corresponding variance, across GWAS by


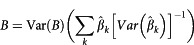


where


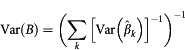


We then obtain a combined *Z*-score for association of the *j*th SNP across GWAS by


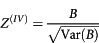


### Simulation study

We have performed a series of detailed simulations to investigate the type I error rates and power of alternative approaches to study-level association testing of a binary phenotype (linear and logistic regression modelling) in the context of fixed-effects meta-analysis (with effective sample size or inverse-variance weighting schemes), summarised in Table 1.

Our first study design consisted of 10 cohorts of a binary phenotype, ascertained from the same population, each comprising of 2000 participants. We considered three scenarios for case–control imbalance, described in Table 2, such that the meta-analysis comprised a total of 10 000 cases and 10 000 population controls: (i) no imbalance (1:1 ratio in each cohort); (ii) moderate imbalance (variable ratio of 3:1 to 1:3 across cohorts); and (iii) extreme imbalance (variable ratio of 19:1 to 1:19 across cohorts). For each scenario, we investigated models of association parameterised according to: (i) the risk allele frequency (RAF) of the causal SNP, denoted *q*; and (ii) the allelic OR for the risk allele, denoted *ψ*.

For each model, we generated 10 000 replicates of genotype data for the causal SNP in the study participants. For each replicate, genotypes were simulated in the required numbers of cases and controls in each cohort, according to the causal SNP RAF and allelic OR, and assuming Hardy–Weinberg equilibrium. Specifically, genotypes in cases and controls were simulated from a multinomial distribution, with probabilities given by


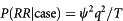



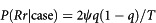



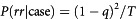



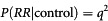



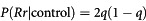



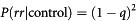


where *R* denotes the risk allele and 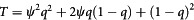.

To assess the impact of confounders on the alternative analysis strategies, we also simulated a binary covariate for each individual from a Bernoulli distribution, taking the value 1 in cases with probability 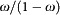 and 0 otherwise, and taking the value 1 in controls with probability 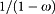 and 0 otherwise.

We also investigated the impact of population stratification on the alternative analysis strategies. Within each cohort, cases and controls were ascertained from sub-population A with probabilities *θ* and (1−*θ*), respectively, and were otherwise ascertained from sub-population B. The RAFs in sub-populations A and B were assumed to be 0.4 and 0.6, respectively, and used to generate genotypes at the causal SNP under Hardy–Weinberg equilibrium, from a multinomial distribution, as defined above. For each individual, we then simulated genotype data for 1000 additional uncorrelated SNPs, assuming Hardy–Weinberg equilibrium, and independent of case–control status, from a multinomial distribution. For each SNP, we assumed minor allele frequencies of 0.2 and 0.8, respectively, in sub-populations A and B. Genotypes at the 1000 SNPs were then used to construct the GRM within each cohort.

Our second study design consisted of two cohorts of a binary phenotype, ascertained from the same population. The first cohort consisted of 1000 cases and 1000 controls. The second cohort represented a large biobank of 100 000 individuals, within which we investigated the impact of the extent of case–control imbalance on the meta-analysis. For each scenario, we assumed a causal SNP RAF of 0.5 and an allelic OR of 1.25, and generated 10 000 replicates of genotype data for the causal SNP in the study participants. For each replicate, genotypes were simulated in the required number of cases and controls in the two cohorts, assuming Hardy–Weinberg equilibrium, from a multinomial distribution, as described above.

For both study designs, we used a linear Wald test, implemented in EPACTS, to obtain parameter estimates and association *P*-values under a linear regression model (no random effects) within each cohort for each replicate. To obtain parameter estimates under a logistic regression model (no random effects) within each cohort, we used a Firth bias-corrected likelihood ratio test, also implemented in EPACTS, which has been demonstrated to be more robust to case–control imbalance than Wald or score statistics for binary outcomes.^13^ To obtain parameter estimates under a LMM (random effects for GRM) within each cohort, we used EMMAX,^1^ also implemented in EPACTS. We combined summary statistics through fixed-effects meta-analysis with effective sample size and inverse-variance weighting using METAL^14^ and GWAMA,^15^ respectively.

Across all scenarios, each test of association, after meta-analysis, was evaluated at nominal significance thresholds of *P*<0.05 and *P*<0.01, and at the traditional genome-wide standard of *P*<5 × 10^−8^. For estimated allelic effect sizes on the log-odds scale (from the logistic regression model and after conversion from the linear regression model), we also evaluated bias and mean square error (MSE).

## Results

### No population stratification or confounders

We first considered the properties of fixed-effects meta-analysis of association summary statistics obtained from linear and logistic regression models *without* random effects for the GRM and for simulations generated in the absence of structure or confounders. Supplementary Figure S1 presents the type I error rate (at a nominal 5% significance threshold) of each of the analytical strategies considered (Table 1) for an SNP with RAF in the range of 1–50%. For all frequencies investigated, the type I error rate was consistent with the nominal significance threshold of *P*<0.05, irrespective of the analytical approach and the extent of case–control imbalance.

**Figure 1:**
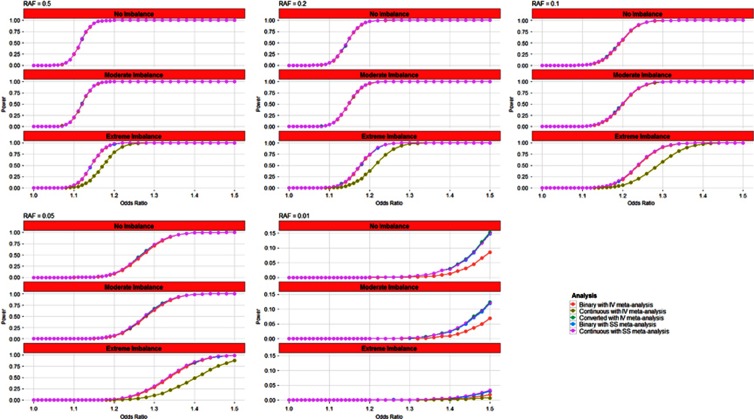
Power to detect association (at genome-wide significance, *P*<5 × 10^−8^) of a binary phenotype with a causal SNP, in the absence of population stratification or confounders, using alternative meta-analysis strategies for summary statistics obtained from linear and logistic regression models without random effects for the GRM (Table 1). Results are presented as a function of the allelic OR, for a causal SNP with RAF in the range of 1–50% and for variable extent of case–control imbalance (defined in Table 2).

Figure 1 presents the power (at genome-wide significance) of each of the analytical strategies considered (Table 1), as a function of the allelic OR, for an SNP with RAF in the range of 1–50%. There is no appreciable difference in power between the five approaches unless there is extreme case–control imbalance. In this extreme imbalance setting, the power of the meta-analysis under inverse-variance weighting of effect sizes from the linear model (without conversion to the log-odds scale) is substantially lower compared with that for the other approaches. However, we also observe a loss in power of the meta-analysis under inverse-variance weighting of effect sizes from the logistic regression model for rare SNPs (RAF 1%), irrespective of the extent of case–control imbalance, which has not been reported previously. We observe the same pattern of results at less stringent significance levels (Supplementary Figure S2), with the inverse-variance weighting of effect sizes from the linear model (without conversion to the log-odds scale) being substantially less powerful when there is extreme case–control imbalance.

Supplementary Figures S3 and S4 present the bias and MSE of the estimated allelic OR after meta-analysis under the inverse-variance weighting of effect sizes from the logistic regression model and the linear regression model after conversion to the log-odds scale. Results are presented as a function of the allelic OR. There is minimal difference in both metrics between the two meta-analysis strategies. However, for rare SNPs (RAF 1%), the meta-analysis under inverse-variance weighting of effect sizes from the logistic regression model underestimates the allelic OR, irrespective of case–control imbalance, explaining the reduction in power of this strategy that was observed above.

### Impact of a confounding variable in the absence of population stratification

We next considered the properties of fixed-effects meta-analysis of association summary statistics obtained from linear and logistic regression models *without* random effects for the GRM and for simulations generated in the absence of structure, but where the binary phenotype was also correlated with a confounding variable. We assumed a causal SNP with RAF 50% and an allelic OR of 1.15 for the binary phenotype. Supplementary Figure S5 presents the power (at genome-wide significance) of each of the five analytical strategies considered (Table 1), as a function of the relative risk of the confounding variable, defined by 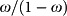. As expected, there is a general decline in power to detect association across analytical strategies as the relative risk of the confounder of the binary phenotype increases. However, as demonstrated by the simulations in the absence of confounders, the inverse-variance weighting of effect sizes from the linear model (without conversion to the log-odds scale) was less powerful when there is extreme case–control imbalance.

Supplementary Figure S5 also presents the bias and MSE of the estimated allelic OR after meta-analysis under the inverse-variance weighting of effect sizes from the logistic regression model and the linear regression model after conversion to the log-odds scale. Results are presented as a function of the relative risk of the confounding variable. Irrespective of the case–control imbalance, the estimated allelic OR after conversion to the log-odds scale becomes increasingly biased (underestimated) as the relative risk of the confounding variable increases, although power is not affected.

### Impact of population stratification

We then considered the properties of fixed-effects meta-analysis of association summary statistics obtained from linear regression models, with and without random effects for the GRM and for simulations generated in the presence of population stratification (cases and controls ascertained from sub-populations A and B). Supplementary Figure S6 presents the type I error rate (at a nominal 5% significance threshold) of each analytical strategy considered (Table 1) as a function of the probability, *θ*, that a case is ascertained from sub-population A. Irrespective of the extent of population stratification, the type I error rate was consistent with the nominal significance threshold of *P*<0.05 for any fixed-effects meta-analysis strategy using the linear model with random effects for the GRM. However, as expected, type I error rates became increasingly inflated as the extent of population stratification was elevated for all fixed-effects meta-analysis strategies using the linear model without a random effect for the GRM.

**Figure 2:**
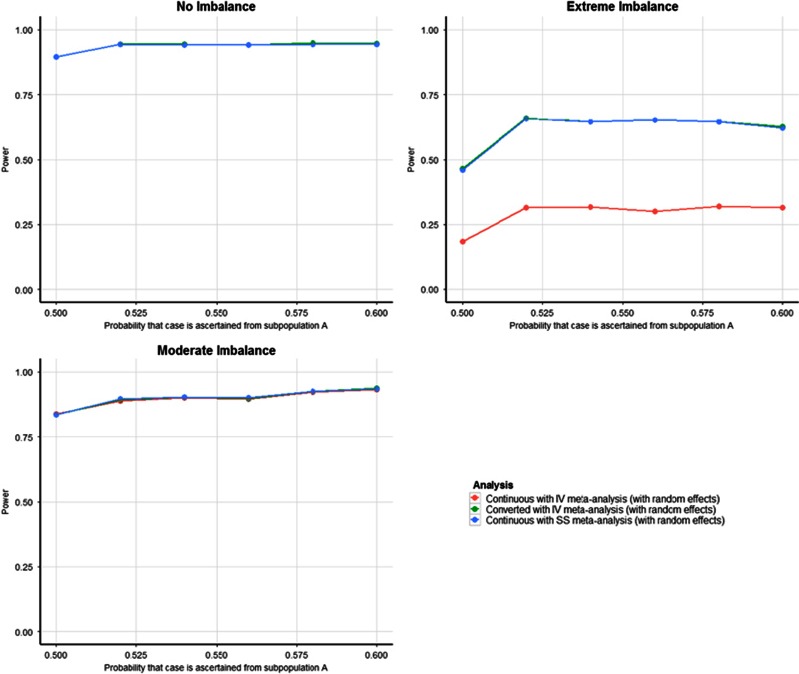
Power to detect association (at genome-wide significance, *P*<5 × 10^−8^) of a binary phenotype with a causal SNP, in the presence of population stratification (cases and controls ascertained from sub-populations (A and B), using alternative meta-analysis strategies for summary statistics obtained from linear regression models with random effects for the GRM (Table 1). Results are presented as a function of the probability that a case is ascertained from sub-population A, for a causal SNP with allelic OR of 1.15 for the binary phenotype and for variable extent of case–control imbalance (defined in Table 2).

Figure 2 presents the power (at genome-wide significance) of the three fixed-effects meta-analysis strategies that aggregate association summary statistics from the linear model with random effects for the GRM, for a causal SNP with allelic OR of 1.15 for the binary phenotype. There is no appreciable difference in power between the analytical strategies, unless there is extreme case–control imbalance. In this extreme imbalance setting, the power of the meta-analysis under inverse-variance weighting of effect sizes from the linear model (without conversion to the log-odds scale) is substantially lower compared with that for the other approaches. The difference in power between these approaches is consistent, irrespective of the extent of population stratification.

### Impact of inclusion of a population biobank with extreme case–control imbalance

**Figure 3:**
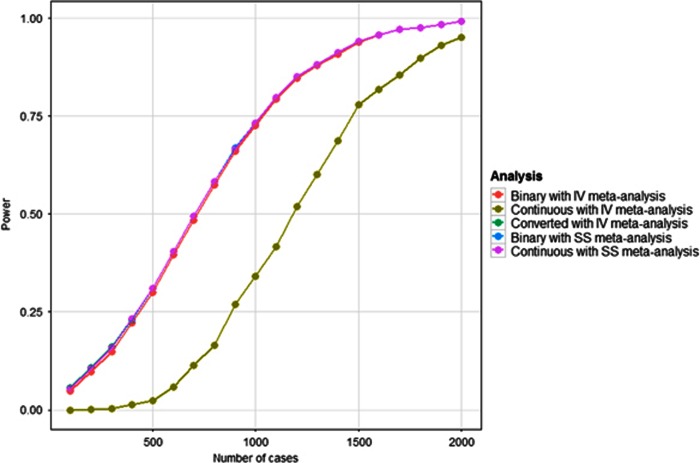
Power to detect association (at genome-wide significance, *P*<5 × 10^−8^) of a binary phenotype with a causal SNP, in the absence of population stratification or confounders, using alternative meta-analysis strategies for summary statistics obtained from linear and logistic regression models without random effects for the GRM (Table 1). Association summary statistics were aggregated from a population biobank of 100 000 participants with extreme case–control imbalance and a balanced case–control study of 2000 participants. Results are presented for a causal SNP with RAF 50% and an allelic OR of 1.25, as a function of the number of cases in the population biobank.

Finally, we considered the properties of fixed-effects meta-analysis of association summary statistics obtained from linear and logistic regression models without random effects for the GRM, for simulations generated in the absence of structure. In these simulations, association summary statistics were aggregated from a population biobank of 100 000 participants with extreme case–control imbalance and a balanced case–control study of 2000 participants. Figure 3 presents the power (at genome-wide significance) of each of the analytical strategies considered (Table 1), for a causal SNP with RAF 50% and an allelic OR of 1.25, as a function of the number of cases in the population biobank. As reported above, in this extreme imbalance setting, the power of the meta-analysis under inverse-variance weighting of effect sizes from the linear model (without conversion to the log-odds scale) is substantially lower compared with that for the other approaches. The difference in power reduces as the extent of the imbalance in the biobank decreases (i.e. the proportion of cases increases), and thus has most detrimental impact for rare diseases.

## Discussion

We have presented simulations to evaluate the utility of linear models in the context of meta-analysis of GWAS of binary phenotypes. Our results highlight that the extent of case–control imbalance across studies can have a major impact on the performance of a linear regression model. We have demonstrated that, for extreme imbalance, meta-analysis under inverse-variance weighting of allelic effect estimates from a linear regression model results in a substantial reduction in power, unless they are first converted onto the log-odds scale. This is of particular importance because existing, widely used software^16^ for the meta-analysis of association summary statistics from LMMs implements inverse-variance weighting of allelic effect estimates *without* conversion to the log-odds scale.

For a binary phenotype, under a linear regression model, the standard error of an allelic effect estimate is dependent on multiple factors, including allele frequency, total sample size, OR and variance of the trait. For a fixed total sample size, the variance of the trait (and thus standard error of the allelic effect estimate) decreases as the case–control imbalance becomes more extreme. However, the power to detect association with the binary phenotype is less in imbalanced studies, and they should, in fact, be given less weight in any meta-analysis. Correction of allelic effect estimates from the linear regression model onto the log-odds scale circumvents this issue by inflating the corresponding standard error by a factor that is inversely proportional to the case–control imbalance.

Case–control imbalance is becoming increasingly widespread in GWAS of binary phenotypes, particularly with the availability of large-scale, extensively studied, population-based biobanks, often with linkage to electronic medical records.^17, 18, 19, 20^ The utility of linear models in these extremely imbalanced case–control designs has not been previously studied in the context of meta-analysis. Crucially, our investigation highlights that linear models can be used for meta-analysis of GWAS of binary phenotypes, without loss of power, even in the presence of extreme case–control imbalance, provided that one of the following schemes is used: (i) effective sample size weighting of *Z*-scores or (ii) inverse-variance weighting of allelic effect sizes after conversion onto the log-odds scale.

Our simulations demonstrate that meta-analysis of association summary statistics for binary phenotypes from LMMs is robust to population stratification, even in the presence of extreme case–control imbalance. However, it is important to note that this conclusion is valid only when population stratification does not lead to violation of the LMM assumption of homoscedasticity, for which residual variances are constant, irrespective of covariates.^21, 22^ Heteroscedasticity can occur in the presence of population stratification, for example, when strata have variable case–control imbalance or heterogeneous disease risk. Under these circumstances, LMMs are valid only for variants that have similar RAFs across strata, such that there is only weak confounding due to structure. Otherwise, computationally efficient software will be required to implement logistic mixed models on the scale of the whole genome.

## Figures and Tables

**Table 1:** Summary of approaches to study-level association testing of a binary phenotype

*Study-level analysis*	*Random effects?*	*Summary statistic*	*Meta-analysis weighting*	*Meta-analysis summary statistic(s)*
Logistic regression	No	*P*-value	Effective sample size	*P*-value
Logistic regression	No	Allelic effect on log-odds scale	Inverse variance	*P*-value and effect size on log-odds scale
Linear regression	No	*P*-value	Effective sample size	*P*-value
Linear regression	No	Alelic effect on linear scale	Inverse variance	*P*-value and effect size on linear scale
Linear regression	No	Allelic effect converted to log-odds scale	Inverse variance	*P*-value and effect size on log-odds scale
Linear regression	GRM	*P*-value	Effective sample size	*P*-value
Linear regression	GRM	Allelic effect on linear scale	Inverse variance	*P*-value and effect size on linear scale
Linear regression	GRM	Allelic effect converted to log-odds scale	Inverse variance	*P*-value and effect size on log-odds scale

Abbreviation: GRM, genetic relationship matrix.

**Table 2:** Summary of case–control counts in each cohort for alternative imbalance scenarios considered in the simulation study

*Cohort*	*No imbalance*		*Moderate imbalance*		*Extreme imbalance*	
	*Cases*	*Controls*	*Cases*	*Controls*	*Cases*	*Controls*
1	1000	1000	600	1400	100	1900
2	1000	1000	700	1300	300	1700
3	1000	1000	800	1200	500	1500
4	1000	1000	900	1100	700	1300
5	1000	1000	1000	1000	900	1100
6	1000	1000	1000	1000	1100	900
7	1000	1000	1100	900	1300	700
8	1000	1000	1200	800	1500	500
9	1000	1000	1300	700	1700	300
10	1000	1000	1400	600	1900	100
Total	10 000	10 000	10 000	10 000	10 000	10 000
