# Supplementary material for: Guidance for the utility of linear models in meta-analysis of genetic association studies of binary phenotypes
Source: Eur J Hum Genet. 2016 Nov 16;25(2):240–5. doi: 10.1038/ejhg.2016.150 (PMC5237383; doi:10.1038/ejhg.2016.150)
Supplement: Supplementary Figures [file ejhg2016150x1.pdf]

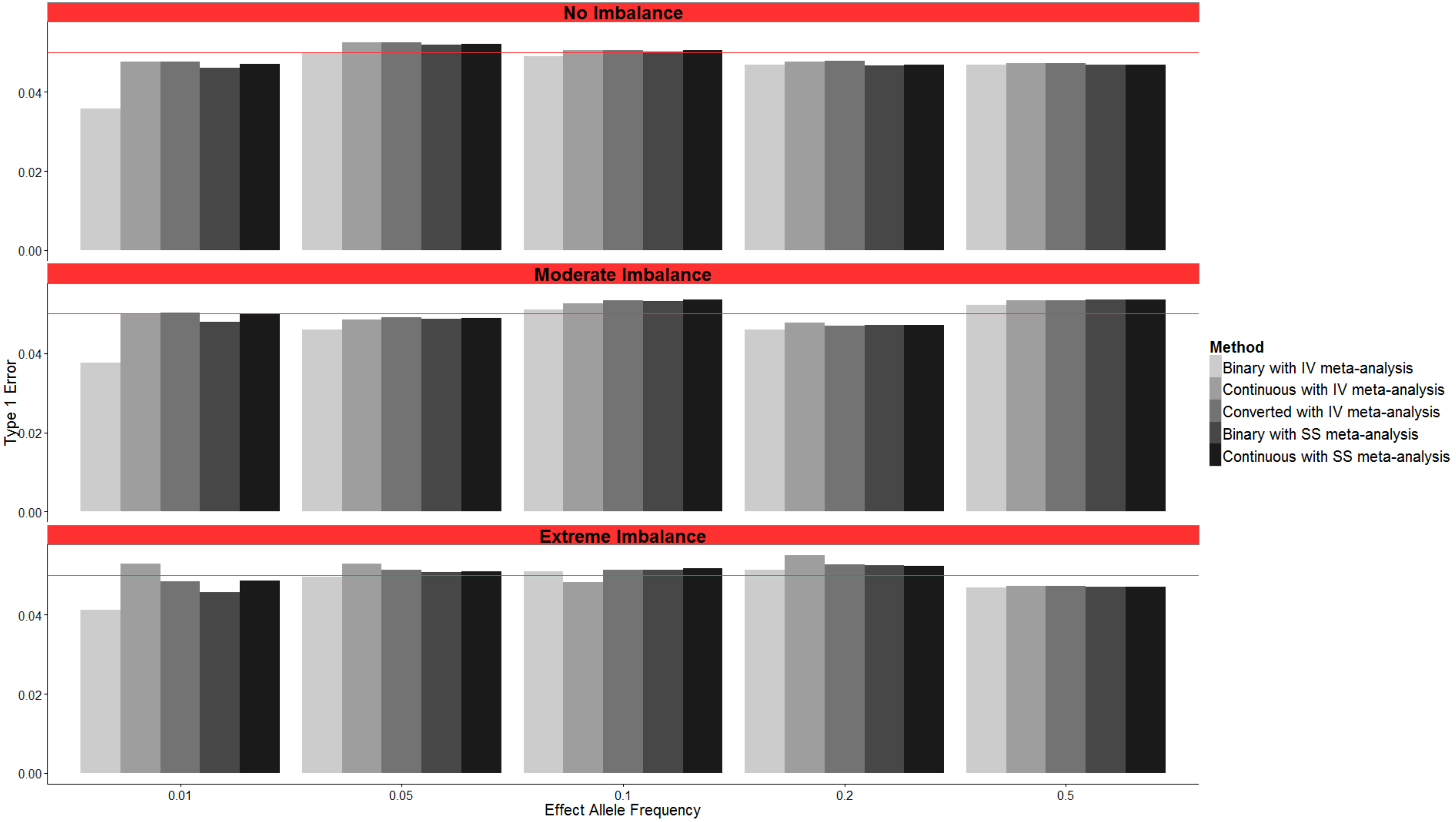

Supplementary Figure S1. Type I error rates to detect association (p<0.05) of a binary phenotype with a causal SNP, in the absence of population stratification or confounders, using alternative meta-analysis strategies for summary statistics obtained from linear and logistic regression models without random effects for the GRM (**Table 1**). Results are presented for a SNP with MAF in the range of 1-50%, and for variable extent of case-control imbalance (defined in **Table 2**).

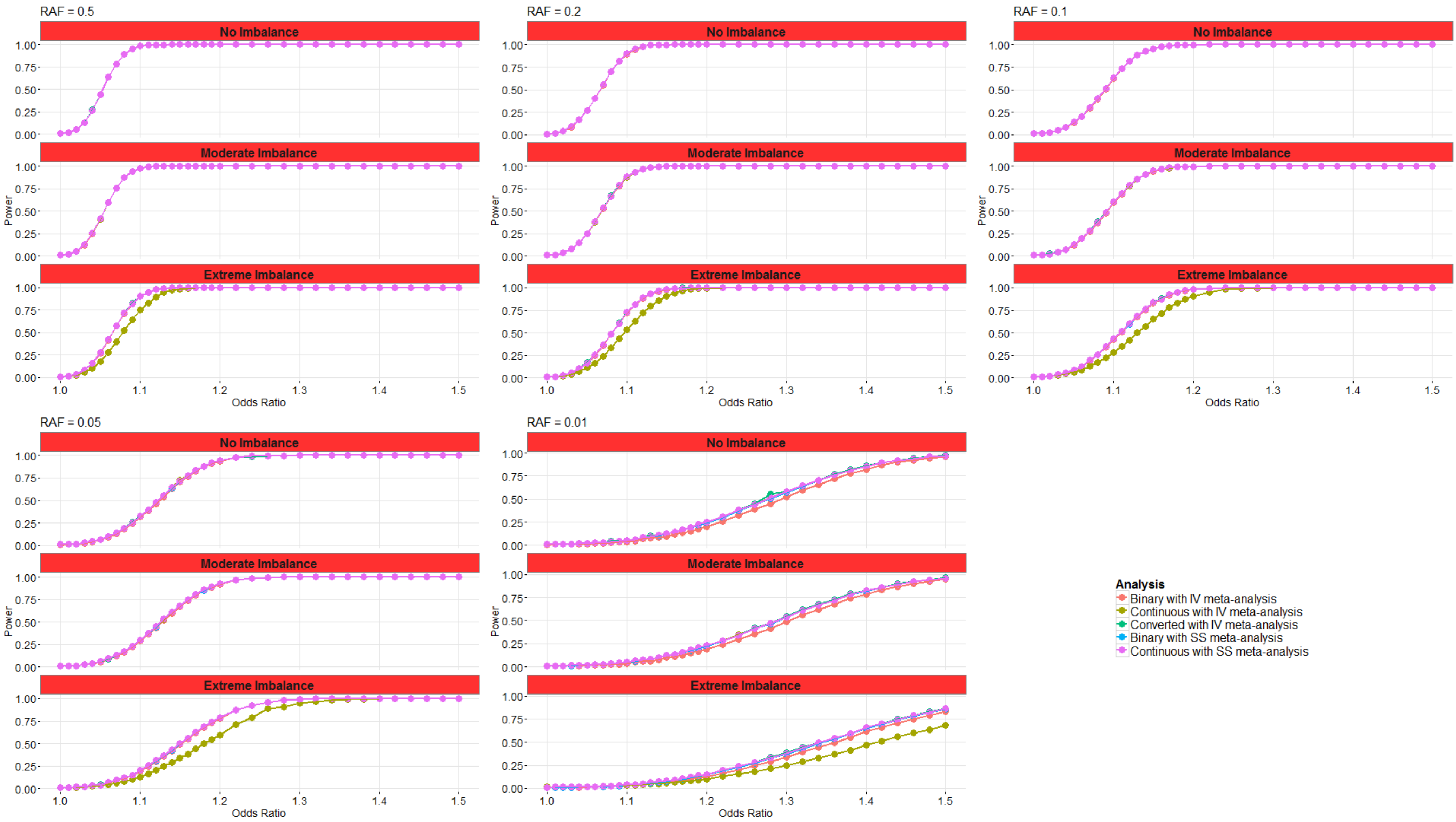

Supplementary Figure S2. Power to detect association (*p*<0.01) of a binary phenotype with a causal SNP, in the absence of population stratification or confounders, using alternative meta-analysis strategies for summary statistics obtained from linear and logistic regression models without random effects for the GRM (**Table 1**). Results are presented as a function of the allelic OR, for a causal SNP with RAF in the range of 1-50%, and for variable extent of case-control imbalance (defined in **Table 2**).

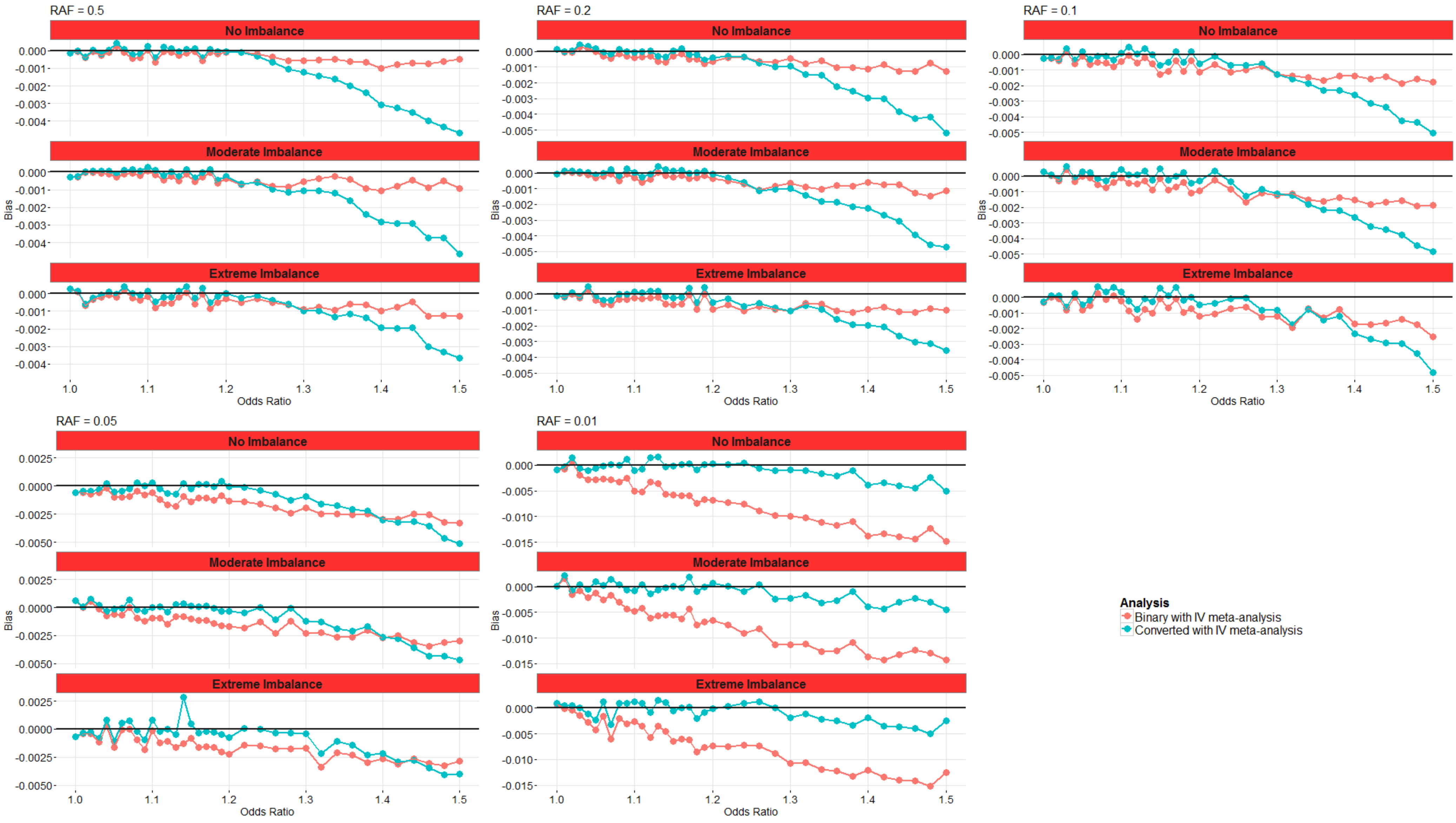

Supplementary Figure S3. Bias of the estimated allelic OR after meta-analysis under the inverse-variance weighting of effect sizes from the logistic regression model and the linear regression model after conversion to the log-odds scale. Results are presented as a function of the allelic OR, for a causal SNP with RAF in the range of 1-50%, and for variable extent of case-control imbalance (defined in **Table 2**).

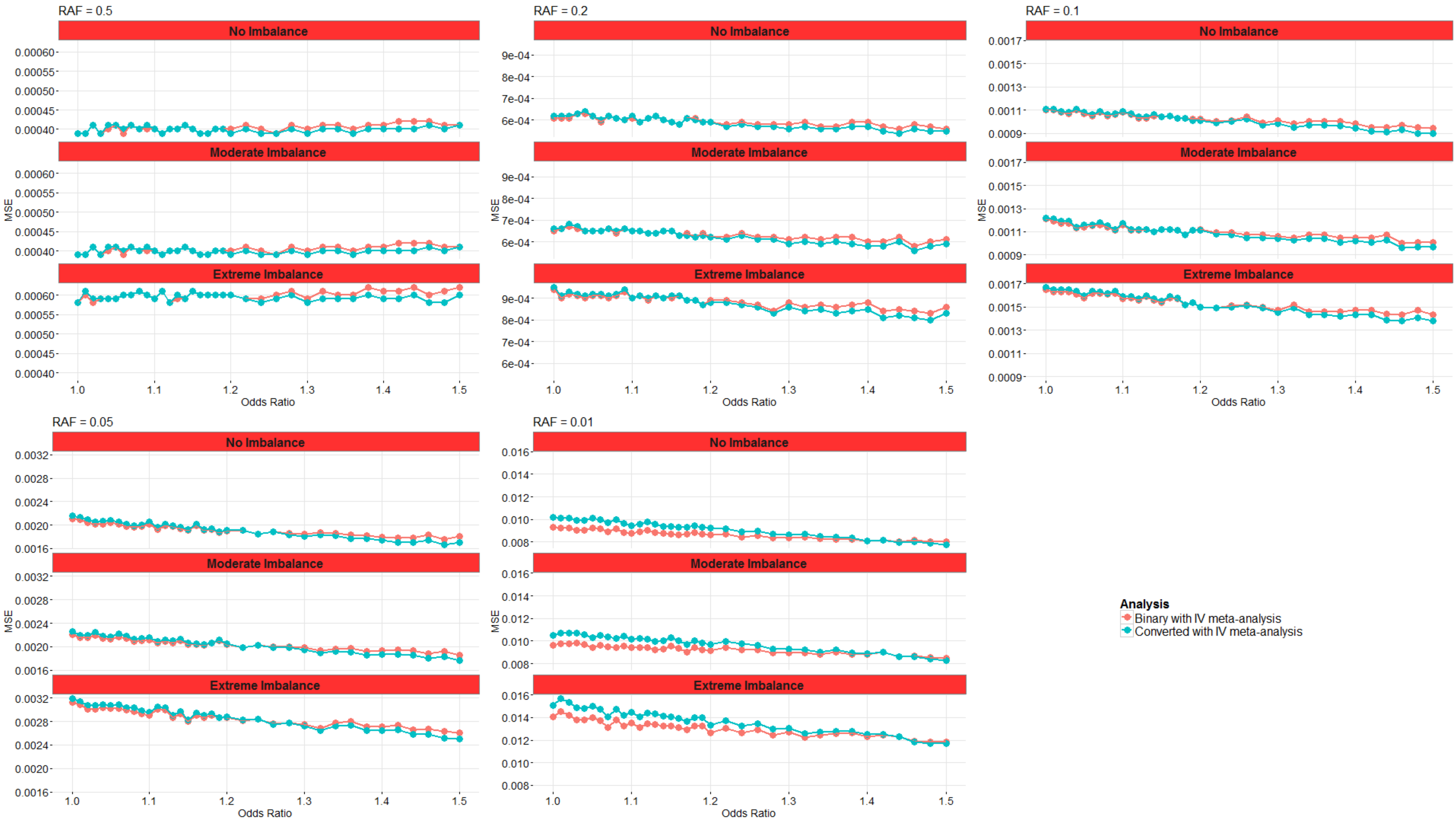

Supplementary Figure S4. MSE of the estimated allelic OR after meta-analysis under the inverse-variance weighting of effect sizes from the logistic regression model and the linear regression model after conversion to the log-odds scale. Results are presented as a function of the allelic OR, for a causal SNP with RAF in the range of 1-50%, and for variable extent of case-control imbalance (defined in **Table 2**).

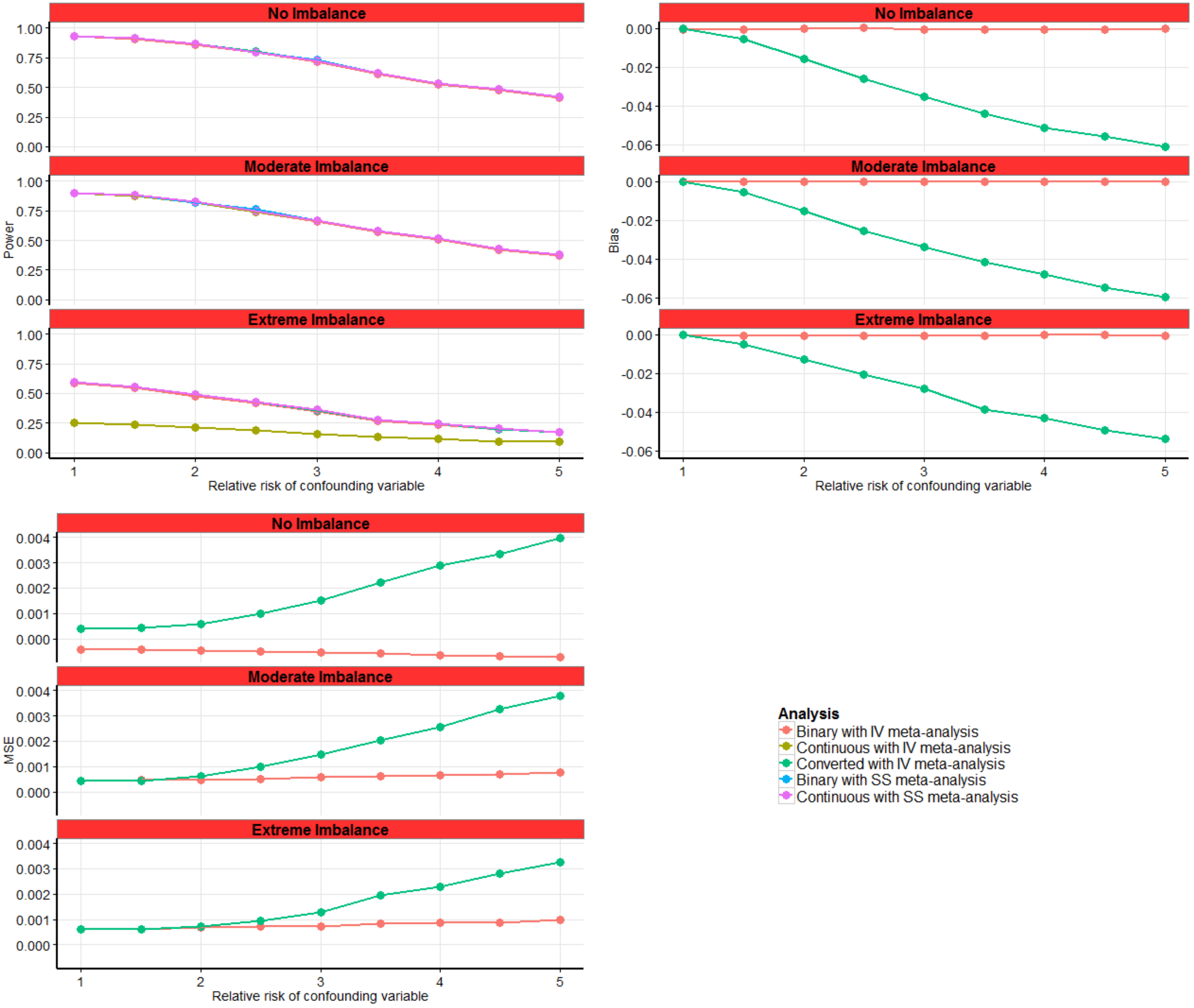

Supplementary Figure S5. Power to detect association (at genome-wide significance, $p<5\times10^{-8}$) of a binary phenotype with a causal SNP, in the absence of population stratification, but with a confounding variable, using alternative meta-analysis strategies for summary statistics obtained from linear and logistic regression models without random effects for the GRM (**Table 1**). Bias of the estimated allelic OR after meta-analysis under the inverse-variance weighting of effect sizes from the logistic regression model and the linear regression model after conversion to the log-odds scale. Results are presented as a function of the relative risk of the confounding variable, for a causal SNP with RAF 50% and allelic OR of 1.15, for variable extent of case-control imbalance (defined in **Table 2**).

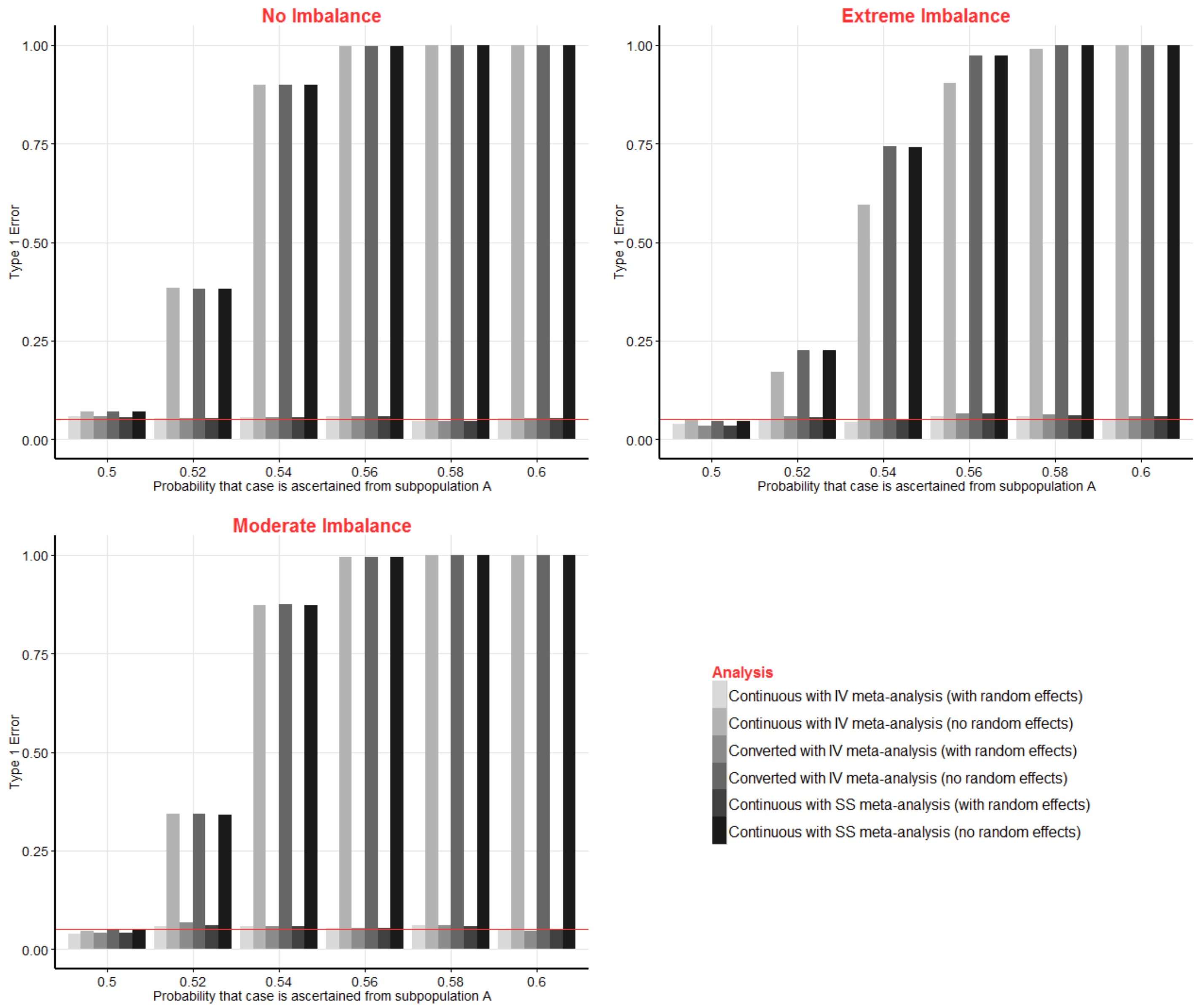

Supplementary Figure S6. Type I error rates to detect association (p<0.05) of a binary phenotype with a causal SNP, in the presence of population stratification, using alternative meta-analysis strategies for summary statistics obtained from linear regression models, with and without random effects for the GRM (**Table 1**), as a function of the probability that cases are ascertained from subpopulation A. Results are presented for a SNP with frequency of 40% in subpopulation A and 60% in subpopulation B, and for variable extent of case-control imbalance (defined in **Table 2**).
